# Umbrella systematic review finds limited evidence that school absence explains the association between chronic health conditions and lower academic attainment

**DOI:** 10.3389/fpubh.2023.1122769

**Published:** 2023-06-09

**Authors:** Matthew A. Jay, David Sanders-Ellis, Ruth Blackburn, Jessica Deighton, Ruth Gilbert

**Affiliations:** ^1^UCL GOS Institute of Child Health, Population, Policy and Practice Research and Teaching Department, University College London, London, United Kingdom; ^2^UCL Institute of Education, Social Research Institute, University College London, London, United Kingdom; ^3^UCL Institute of Health Informatics, University College London, London, United Kingdom; ^4^The Evidence Based Practice Unit, University College London and Anna Freud Centre for Children and Families, London, United Kingdom

**Keywords:** chronic health conditions, school absence, academic attainment, academic achievement, mediation, meta-review

## Abstract

**Introduction:**

Absence from school is more frequent for children with chronic health conditions (CHCs) than their peers and may be one reason why average academic attainment scores are lower among children with CHCs.

**Methods:**

We determined whether school absence explains the association between CHCs and academic attainment through a systematic review of systematic reviews of comparative studies involving children with or without CHCs and academic attainment. We extracted results from any studies that tested whether school absence mediated the association between CHCs and academic attainment.

**Results:**

We identified 27 systematic reviews which included 441 unique studies of 7, 549, 267 children from 47 jurisdictions. Reviews either covered CHCs generally or were condition-specific (e.g., chronic pain, depression, or asthma). Whereas reviews found an association between a range of CHCs (CHCs generally, cystic fibrosis, hemophilia A, end-stage renal disease (pre-transplant), end-stage kidney disease (pre-transplant), spina bifida, congenital heart disease, orofacial clefts, mental disorders, depression, and chronic pain) and academic attainment, and though it was widely hypothesized that absence was a mediator in these associations, only 7 of 441 studies tested this, and all findings show no evidence of absence mediation.

**Conclusion:**

CHCs are associated with lower academic attainment, but we found limited evidence of whether school absence mediates this association. Policies that focus solely on reducing school absence, without adequate additional support, are unlikely to benefit children with CHCs.

**Systematic review registration:**

https://www.crd.york.ac.uk/prospero/display_record.php?RecordID=285031, identifier: CRD42021285031.

## 1. Introduction

On average, children with chronic health conditions (CHCs) are less likely to perform well in school exams than their healthy peers (1–4), a factor which is a particular concern for children and young people (5), their parents (6), teachers (7, 8) and policymakers alike (9–11). One possible mechanism that explains poorer average academic attainment among such children is absence from school related to their CHC. Although school absence is extremely common, it is particularly prevalent among children with CHCs (3), who are more likely to be absent due to illness and healthcare usage (4). Absence from school is assumed to cause lower attainment partly due to the strong association reported in a national analysis by the UK Department for Education (12), which found that every extra day missed from school was associated with lower attainment (13).

**Figure 1:**
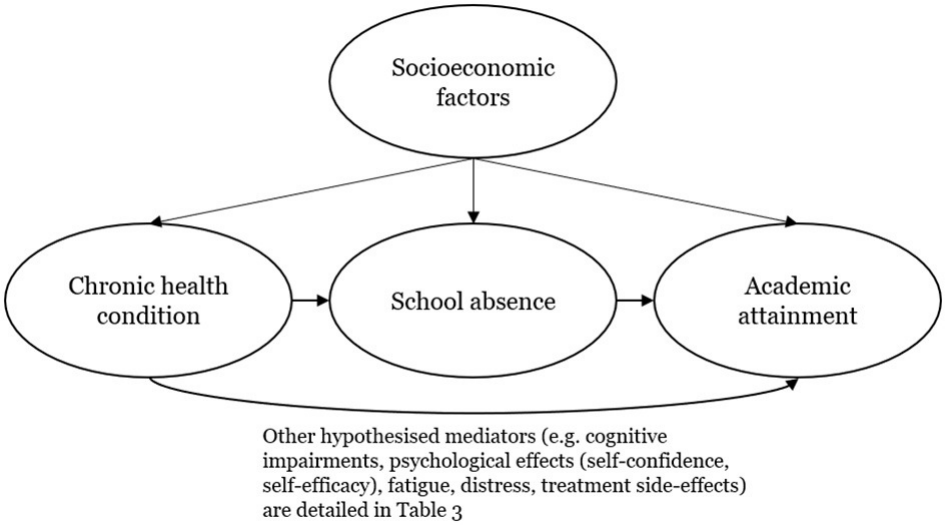
Simplified directed acyclic graph showing hypothesized relationships between chronic health conditions, school absence, socioeconomic confounders, and academic attainment.

Evidence that absence causes lower attainment is critical to guide how schools respond to absence among children with CHCs. CHCs are common: Van Cleave et al. (14) estimated that, between ages 8 and 14 years, the cumulative prevalence of having a parent-reported CHC was between 13% and 27% depending on the year of birth and case definition. The Department for Education study did not explore whether absence caused low attainment, particularly among children with CHCs. Analyses that distinguish whether absence mediates the effect of CHCs on school attainment need to take into account common causes of CHCs, increased absence, and reduced attainment (Figure 1). For example, the health condition itself, or its clinical management, may be a cause of school absence, especially symptom-related absence, and independently, a cause of reduced attainment, particularly for conditions linked to cognitive or behavioral deficits. Socioeconomic factors may also be a common cause of CHCs, absence, and attainment given well-known links between poorer socioeconomic status and health as well as school absence (15) and lower attainment (16).

In England, the Children's Commissioner, an influential official responsible for promoting the views and interests of children, has called for a reduction of school absence to zero percent (11). Official government guidance, reflecting statutory provisions, does not mandate a reduction of absence to zero but instead emphasizes the legal duty incumbent on parents to ensure that their child, when enrolled in a school, attends school every day. The guidance also recognizes that children with long-term health conditions face additional barriers to attendance which must be addressed to ensure that they can enjoy their right to full-time education (13). Policies aimed at reducing absence to zero could benefit children with CHCs if school absence is the mechanism, or mediator, through which CHCs reduce academic attainment. However, if the relationship between absence and attainment is because CHCs, or other factors linked to CHCs (such as socioeconomic circumstances), are a common cause of increased absence and reduced attainment, then focusing on reducing absence may not be helpful and could be harmful, especially if not accompanied by adequate resources to meet the needs of children with CHCs in schools.

Policies that focus on reducing absence can have adverse consequences for children and young people with a CHC. In preparation for our programme of work on CHCs and education, we consulted a group of 22 children and young people with and without CHCs (November 2021) and a separate group of six parents of affected children (May 2022). Some reported feeling alienated by school practices around attendance and discipline, such as strict behavior policies and parents and children being labeled a “problem” in relation to frequent absence. Others mentioned the pressure to explain and justify their illness, evidence a diagnosis, which is sometimes not possible, and to return to school before full recovery. Some students and parents felt there was inflexibility from the school, such as setting arbitrary attendance targets, and lack of understanding of learner needs leading to difficulties in the classroom. These potential harms underline the need for clear evidence of the benefit of absence reduction policies on student attainment, health, and wellbeing.

This umbrella review (i.e., a systematic review of systematic reviews) aimed to inform policy responses to school absence by reviewing the evidence for absence being the mechanism mediating the association between CHCs and academic attainment. We (a) reviewed evidence from systematic reviews that presented evidence of an association between CHCs and academic attainment and (b) we examined the subset of studies that tested whether absence mediated the association between CHCs and academic attainment by exploring what the results were and how the studies accounted for confounding. We report results separately for different CHCs because the causes, treatments, and effects on academic attainment may differ. For example, some conditions or treatments may cause direct cognitive impairments such as central nervous system tumors and their treatment, whereas other conditions, such as asthma, would not.

## 2. Materials and methods

### 2.1. Protocol registration

We, *a priori*, developed a protocol and registered it with the PROSPERO register of systematic reviews (CRD42021285031). This review is reported according to the PRISMA statement (Supplementary File 1).

### 2.2. Inclusion criteria

We included any systematic review of comparative, quantitative studies of any design that quantified the association between a CHC in childhood and academic attainment. CHC status (different types or levels of severity or CHC vs. none) had to be considered as the exposure and a measure of academic attainment as the outcome. Academic attainment could be measured based on these factors: school grades, administered standardized tests of, for example, reading or mathematical ability, whether children graduated from compulsory schooling, or whether they experienced grade retention (i.e., “held back” a year). We included these measures whether they were labeled as academic attainment, achievement, or some other construct but refer to them in this study collectively as academic attainment. Reviews were excluded if they were not a published systematic review (e.g., a narrative review or conference abstract only and no associated, published systematic review could be found and the authors could not be reached) or the review was not peer-reviewed.

### 2.3. Information sources and search strategy

On 27 September 2021, one author (MAJ) searched MEDLINE, Embase, and PsycINFO *via* Ovid. MAJ also searched the Education Resource Information Center (ERIC) and the Education Database, both *via* ProQuest, on 14 October 2021. On 22 March 2022, MAJ further searched all the above databases for reviews related to chronic gastroenterological conditions as keywords for these were omitted from our initial searches. Full search terms and numbers of hits are available in Supplementary File 2. In summary, titles and abstracts were searched for keywords first relating to CHCs (using both generic terms such as “chronic condition^*^” and specific conditions such as “asthma”) combined with a detailed list of terms for educational outcomes adapted from Caird et al. (17). MAJ and another author (DSE) also scanned reference lists of included reviews for further eligible systematic reviews, and we used Google Scholar to search for systematic reviews citing the systematic reviews included. No language, country, or date limitations were specified.

### 2.4. Selection process

The results from the database searches were downloaded as ^*^.RIS files and imported into the Mendeley reference management software. Using a Google Form, which was piloted on the first 50 records, two authors (MJ and DS-E) independently screened all titles and abstracts for eligibility. The same two authors then independently screened the full texts of potentially eligible studies, including those identified from the reference list and Google Scholar searches. Disagreements were resolved through discussion with reference to a third reviewer (RG) necessary in one instance.

### 2.5. Data extraction and effect measures

One author (MJ) examined all included studies and extracted into Microsoft Excel the following information about each review: its authors, year of publication, outcomes studied, language and year limits, inclusion criteria, the number of studies examining academic attainment out of the total number of studies included, whether the review authors hypothesized or assumed that absence was a mediator in any association between CHCs and academic attainment, and any other hypothesized mediators. Whether a particular factor was considered a mediator by the review authors was determined from the language used throughout each manuscript indicating a hypothesized or assumed causal relationship among the CHC, absence, and academic attainment. The review authors did not have to use terms such as “mediator” or “mediation.”

From each review, the following were also extracted for the subset of studies examining academic attainment (i.e., ignoring studies included in the review that examined other outcomes, such as receipt of special educational services): sample sizes of the studies, their countries, comparison groups, overall results on academic attainment, whether the study empirically tested whether the absence was a mediator and, if so, the results of that test. Where mediation was analyzed, we also collected details about the analysis used including statistical methods used, and whether the analyses were adjusted for confounding variables (and, if so, which variables).

### 2.6. Risk of bias assessment

Two authors (MJ and DS-E) both independently used the Risk of Bias in Systematic Reviews (ROBIS) tool to assess the risk of bias in the reviews. The ROBIS assesses systematic reviews on four domains (study eligibility criteria, identification and selection of studies, data collection and study appraisal, and synthesis and findings) and results in an overall assessment of the risk of bias. For each domain and the overall assessment, a review can be rated as low, high, or unclear risk. Disagreements were resolved through discussion.

### 2.7. Synthesis

The systematic reviews were described narratively in terms of target conditions, outcomes, language and year limits, inclusion criteria, number of studies included, sample sizes of included studies, comparison groups, overall results on attainment, and risk of bias. The individual studies within the reviews were described quantitatively in terms of the conditions studied, countries, years, and whether the sample was drawn from a clinical or community population. As some individual studies were included in more than one review, it was necessary to deduplicate the database of individual studies prior to analysis. A study was only considered a duplicate if it was cited in more than one systematic review for the same condition. For example, the individual study by Austin et al. (18), which examined asthma and epilepsy, was cited by both Milton et al. (19) (on asthma) and Wo et al. (20) (on epilepsy). Since different analyses were used in reviews of different conditions, this was not considered a duplicate. Where a study was cited by a review of CHCs generally and by a more specific review (e.g., Fletcher (21), which was cited by Esch et al. (2) (mental disorders) and Hale et al. (22) and McKinley Yoder and Cantrell (1) (both of CHCs generally), the study was counted under the more specific condition.

Finally, we identified the hypothesized causal mechanisms proposed by the review authors. We calculated the number and proportion of reviews within which school absence was a hypothesized or assumed mediator. We calculated the number and proportion of individual studies within which absence mediation was empirically tested, and we present the results of these analyses separately as well as their strengths and limitations in relation to study design and statistical analysis.

## 3. Results

### 3.1. Review selection

**Figure 2:**
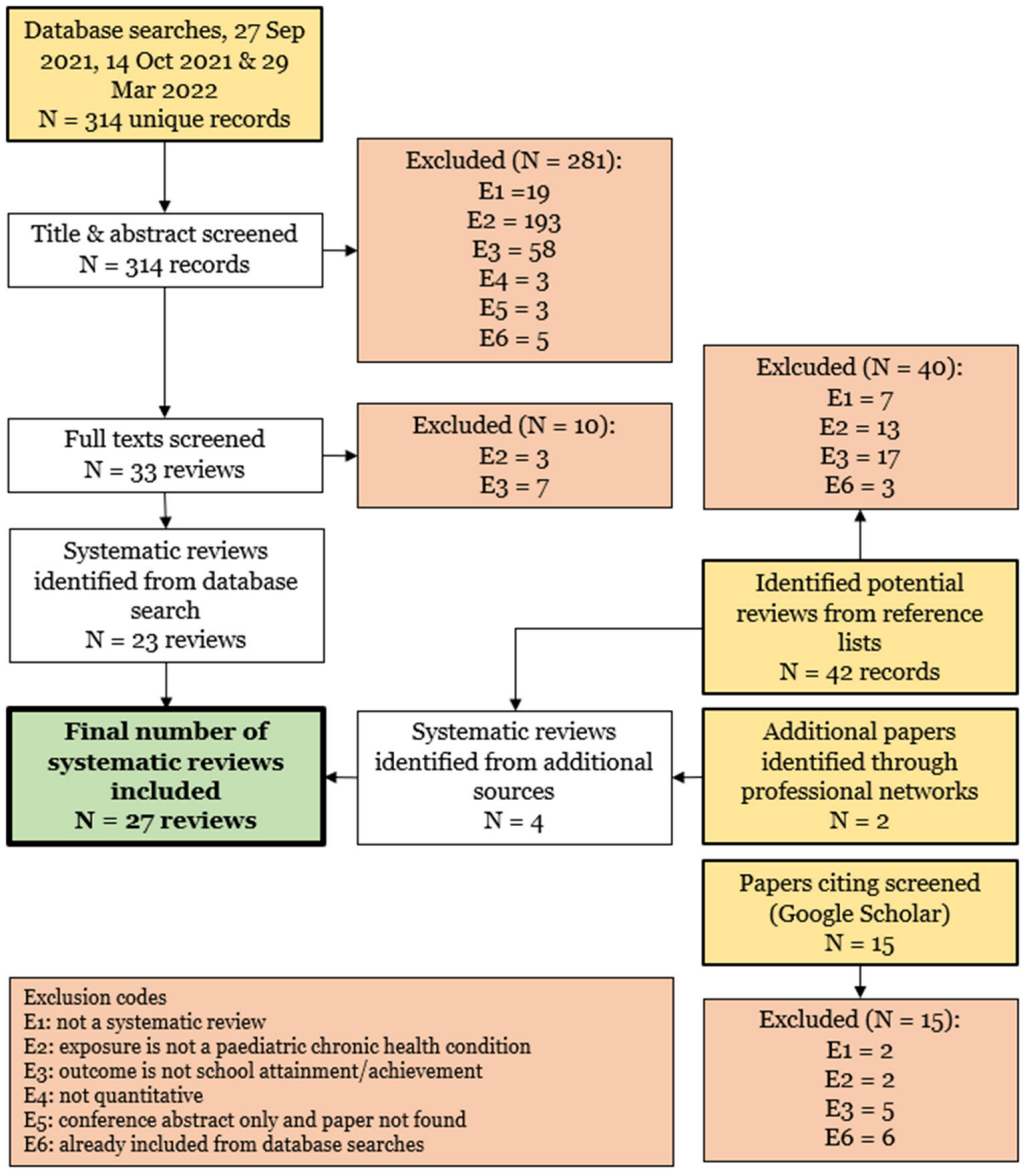
Flow diagram showing study identification and selection.

Our database searches identified 314 unique records, of which 281 were excluded by initial screening (Figure 2). Of the 33 full texts screened, 10 were excluded, resulting in 23 reviews identified from database searches. An additional four reviews were identified from reference lists (two reviews) and professional networks (two reviews). No additional reviews were identified from Google Scholar forward citation searches. The final number of reviews included was therefore 27 (1, 2, 17, 19, 20, 22–43).

### 3.2. Characteristics of reviews and studies

An overview of the reviews, their results on academic attainment, and the results on absence mediation are given in Table 1. Further details on the reviews (health conditions, outcomes, language limits, years covered, inclusion criteria, number of studies, countries, sample sizes, comparison groups, and overall results) are given in Supplementary File 3. Further information on absence mediation is presented below.

**Table 1:** Overview of reviews and studies on chronic health conditions, school absence, and school achievement or attainment.

**Condition**	**# Reviews**	# Studies^*^	**Overall review conclusions on association with academic attainment**	**# Reviews hypothesizing absence mediation**	**Mediation by school absence**
***Multiple***					
Generic / multiple	3 (1, 22, 33)	41	-ve	3	**1 study: no mediation by absence (asthma and cancer/diabetes/epilepsy [as one variable])**.
Major structural congenital anomalies	1 (27)	31	-ve	1	**1 study: no mediation by absence (orofacial clefts)**.
Mental disorders	1 (2)	35	-ve	0	Not tested.
***Condition-specific***					
Asthma	2 (19, 38)	20	1 review: -ve 1 review: none	2	**2 studies: no mediation by absence**.
Attention problems	1 (37)	15	-ve	0	Not tested.
Cancer	4 (29, 32, 36, 40)	38	-ve for CNS tumors (in all four reviews) Mixed for other cancers (3 reviews)	1	Not tested.
Chronic kidney disease	1 (24)	11	-ve	1	Not tested.
Chronic pain	2 (23, 35)	23	-ve	2	Not tested.
CHD	1 (26)	32	-ve	1	Not tested.
Depression	2 (25, 42)	51	-ve	2	Not tested.
Epilepsy	3 (20, 28, 43)	42	Mixed though -ve for children with poor prognosis	1	Not tested.
Obesity	4 (17, 31, 39, 41)	89	None or minimal, but more evident among girls. +ve in some studies	2	**3 studies: no mediation by absence**.
Type 1 diabetes	2 (30, 34)	13	1 review: none or +ve 1 review: -ve (but weak)	2	Not tested.
**Total**	**27**	**441**		**18 (67%)**	**7 (2%) (none finding mediation by absence)**

Five reviews focused on CHCs in general or multiple CHCs (2, 22, 27, 33). Two of these included any chronic condition (1, 22), one included studies on cystic fibrosis, hemophilia A, end-stage renal disease, or end-stage liver disease (33), one included various major structural congenital anomalies (27), and one included mental disorders (2). The remaining 22 reviews were condition-specific covering asthma (19, 38), attention problems (37), cancer (29, 32, 36, 40), chronic kidney disease (24), chronic pain (23, 35), congenital heart disease (26), depression (25, 42), epilepsy (20, 28, 43), obesity (17, 31, 39, 41), and type 1 diabetes (30, 34). Most reviews (*n* = 21) only included studies written in English (1, 17, 19, 20, 22, 23, 25, 27–34, 38–43). One included studies in English, French, or German (2), one included studies in English or Swedish (35), and four specified no language limits (24, 26, 36, 37). Supplementary File 3 also shows year limits imposed by the reviews, study inclusion criteria, number of studies included per review, their sample sizes, and comparison groups. In some instances, reviews required a healthy comparison group though this was not universal, and some reviews included studies with population norms as the comparator or children with different stages of disease (e.g., children on renal dialysis *vs*. those who had received a renal transplant).

**Figure 3:**
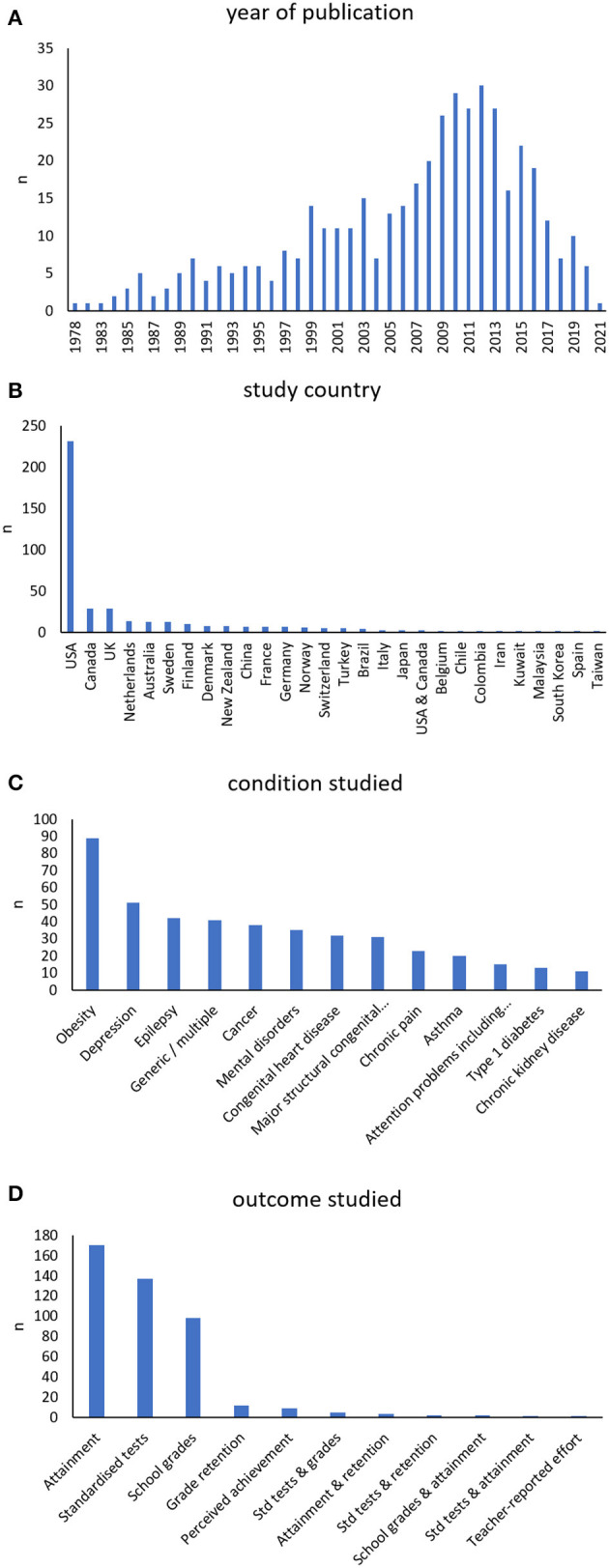
The year of publication **(A)**, study country **(B)**, condition studied **(C)**, and outcome measure used **(D)** in each of the 441 studies included in the systematic reviews. In panel **(B)**, only regions with at least two studies are shown. Regions omitted from the graph with only one study were Austria, Greece, Iceland, Israel, Jamaica, South Korea, Malta, Mexico, Peru, Portugal, Sri Lanka, and Thailand. There were also single studies covering multiple regions: Finland and Sweden, Australia and New Zealand, several high-income countries, Scandinavia, the USA, the UK, and Canada, and the World Mental Health Survey.

Before deduplication, the 27 reviews included 472 studies. After deduplication, there were 441 studies covering a total of 7, 549, 267 children from 47 regions. Of the 441 studies, 268 (61%) drew their samples from community populations (or were analyses of whole-population administrative data or registries) and the remaining 173 studies (39%) used clinical samples. The years of publication of the individual studies, as well as their countries, conditions studied, and outcome measures are shown in Figure 3. Most of the included research was published since the turn of the millennium (Figure 3A) and research from the USA dominated, with 231 (52%) studies from that country (Figure 3B). The top five conditions studied the most were obesity in 89 (20%) studies, followed by depression, epilepsy, chronic conditions generally, and cancer (Figure 3C). Studies used a range of educational measures, most commonly attainment of a particular level of education (170 studies, 39%), followed by administration of standardized tests (137 studies, 31%) and school grades (98 studies, 22%) as shown in Figure 3D. Twelve studies examined grade retention, nine examined perceived achievement, and one used a teacher-reported effort score. The rest of the studies used a mix of attainment, grades, grade retention, and standardized tests.

### 3.3. Risk of bias assessment of reviews

The results from the risk of bias analysis using the ROBIS tool are presented in Supplementary File 4.

### 3.4. Results on achievement or attainment

Most reviews concluded that CHCs were associated with lower academic attainment (Table 1 and Supplementary File 3). Associations between having a CHC and lower academic attainment were reported for CHCs generally, cystic fibrosis, hemophilia A, end-stage renal disease (pre-transplant), end-stage kidney disease (pre-transplant), spina bifida, congenital heart disease, orofacial clefts, mental disorders, depression, and chronic pain (1, 2, 22–27, 33, 35, 42). The results for asthma were mixed with one review (19) concluding that asthma was not associated with lower academic attainment, except possibly severe asthma, whereas in another review (38), five out of eight studies found lower academic attainment in children with asthma. In terms of cancer, association with central nervous system tumors was most persistently observed (29, 32, 36, 40). Evidence for poorer academic attainment among survivors of other cancers was mixed and weaker. Similarly, evidence as to epilepsy was mixed (20, 28, 43) though children with epilepsy with poor prognosis had significantly poorer results in one review (43). Conclusions from the reviews of obesity were that, if there is an association, it is likely not of clinical significance (17, 31, 39, 41). Evidence for type 1 diabetes was mixed and weak (30, 34). Finally, attention problems were associated with lower academic attainment in a review of 15 studies (37), but in 2 studies at low risk of bias, there was no association once IQ and socioeconomic status were adjusted for.

### 3.5. Absence mediation

Table 1 shows that in 18 of the 27 reviews (67%), it was hypothesized that school absence was a mediator. However, this was tested in only 7 of 441 studies (2%) (44–50). Details for these studies, which examined asthma, obesity, orofacial clefts, and cancer/diabetes/epilepsy (analyzed as one, binary variable), on the one hand, and academic attainment, on the other, are shown in detail in Table 2. Comparisons, as detailed in Table 2, were either with children without CHCs or between different levels of severity of CHCs. In six of the seven studies (45–50), multiple regression modeling was used to first adjust for confounding factors, and then additionally adjust for school absence. In all six studies, there was no evidence that school absence was a mediator in the association between each CHC and academic attainment. In the seventh study of orofacial clefts (44), multiple regression was also used, but school absence was adjusted for first, and then confounders were entered into the model. In both steps, the model coefficients were the same. Therefore, from all seven studies, no evidence of absence mediation was found.

**Table 2:** Results of analyses of school absence mediation in the association between chronic health conditions and school achievement or attainment.

**Study [review]**	**Condition, sample size**	**Location (data source)**	**Statistical method**	**Results of mediation analyses**	**Conclusion**	**Strengths**	**Limitations**
Crump et al. (48) [Schneider (38)]	Asthma, 22, 730	San José, California, USA (novel survey linked to school records)	Generalized estimating equations on the odds of achieving “basic or below” on California standard tests. **Step 1**: unadjusted. **Step 2**: adjusted for age, gender, ethnicity, language, grade level, special education, participation in the Free or Reduced Price Lunch program, and parental education. **Step 3**: same as step 2 + school absence.	Step 1: odds of achieving basic or below results were 1.45 times higher (95% CI 1.36, 1.56) among those with any CHC versus no CHC. Step 2 (confounder adjustment): OR 1.25 (1.16, 1.36). Step 3 (further adjusting for absence): OR to 1.22 (1.12, 1.32).	**No evidence of mediation via absence**.	Educational data obtained from school records. Health measured prior to outcomes. Representative of the community.	Parental self-report of child's health condition based on a survey with pre-specified options (e.g., asthma, seizures, any other condition).
Kohen (49) [Schneider (38)]	Asthma, 4, 616	Canada (National Longitudinal Survey of Children and Youth)	Logistic regression of low scores on standardized mathematics and reading tests. **Step 1**: adjusted for age, sex, maternal age, female family headship, maternal education, and household income. **Step 2**: same as step 2 + school absence.	Step1: odds of achieving low scores on maths tests were associated with asthma (low severity: OR 1.39 [1.00, 1.92]; moderate: 1.62 [1.17, 2.25]; severe: 1.62 [1.17, 2.25]) and other chronic conditions (1.75 [1.43, 2.14]) versus no CHC. Step 2: ORs attenuated only minimally (asthma, low 1.36; moderate 1.84, severe 1.59; other conditions 1.72). Results for reading tests were similar.	**No evidence of mediation via absence**.	Educational outcomes based on standardized tests administered in the classroom. Nationally representative.	Asthma measured by reported past-year wheezing or whistling in the chest and regular use of inhalers. Health data and data on school absence reported by the mother or another person (not the child).
Champaloux and Young (50) [Schneider (38) and McKinley Yoder and Cantrell (1)]	Chronic conditions (generic), 6, 795	Canada (National Longitudinal Survey of Children and Youth)	Logistic regression of non-completion of a high school diploma. **Step 1**: adjusted for age, gender, race/ethnicity, parental education, and whether one or two parents in the household. **Step 2**: same as step 1 + school absence.	Step 1: Asthma and cancer/ diabetes/ epilepsy (analyzed as one binary variable) were associated with not completing high school versus no CHC. Asthma: OR 1.63 (1.31, 2.02); cancer/diabetes/epilepsy: 1.96 (1.13, 3.37). Step 2 (absence adjustment): OR 1.66 (1.33, 2.07) and 1.82 (0.99, 3.35), respectively. Heart conditions and other conditions were not associated in step 1 and ORs did not differ when further adjusting for absence.	**No evidence of mediation via absence**.	Health measured prior to educational outcomes. Nationally representative.	Cancer/ diabetes/ epilepsy were entered into the model as one variable, though these may have very different causal relationships with school absence and educational outcomes. Health self- or parent-reported and educational outcome self-reported.
Fitzsimons et al. (44) [Glinianaia et al. (27)]	Orofacial clefts, 3, 253	England, UK (National Pupil Database linked to clefts registry and hospital administrative data)	Logistic regression of achieving expected standard on Key Stage 1 assessments (age 6/7 years). **Step 1**: unadjusted. **Step 2**: adjusted for school absence. **Step 3**: same as step 2 + sex, area-based deprivation, free school meal eligibility.	Step 1: cleft palate only associated with lower odds of achieving the expected standard compared to children with cleft lip only (OR 0.70 [95% CI 0.58, 0.84]). Children with cleft lip and palate also had lower odds (0.70 [0.58, 0.85]). Step 2: Adjusting for absence did not alter this association (cleft palate only OR 0.72 [0.60, 0.88]; cleft lip and palate OR 0.76 [0.63, 0.92]). Step 3 (confounder adjustment): OR 0.64 (0.52, 0.78) and 0.77 (0.63, 0.94), respectively.	**No evidence of mediation via absence**.	Uses all-of-England administrative data. Objective measures of orofacial cleft, absence, and educational outcomes obtained from health and school records. Nationally representative.	Absence adjusted for before control variables. No measure of association against children without orofacial clefts. Limited to early primary school assessments of seven year-old children. At this age, children are teacher-assessed (i.e., no blind marking).
Black et al. (47) [Segal et al. (41)]	Obesity, 4, 983	Australia (Longitudinal Study of Australian Children)	Z-scores on mathematics and literacy tests were examined using ordinary least squares, value added models, and fixed effects models. **Step 1**: unadjusted correlations. **Step 2**: adjusted for 19 variables including age, number of younger/older siblings, rurality, school type, school readiness, teacher's experience in teaching, main language, maternal age, parental education, maternal work status, and household income. **Step 3**: adjust for often or always being absent in the previous month because of not feeling well.	Because associations between obesity and mathematics and literacy scores among girls were non-significant once adjusting for confounders, these analyses were only conducted for boys. For both literacy and mathematics scores, obesity was associated with lower achievement across a range of model specifications, including adjusting for confounders. Further adjusting for absence did not change the coefficient estimates.	**No evidence of mediation via illness-related absence**.	Education outcomes measured by linkage to school records. Child's weight and height measured by a trained interviewer. Nationally representative.	Limited to absence due to not feeling well in the past month (parent-reported), though children with CHCs may be absent for other reasons such as attending appointments and the effects of absence may be longer-lasting.
Sabia (46) [Segal et al. (41) and Caird et al. (17)]	Obesity, 5, 129	USA (National Longitudinal Study of Adolescent Health)	Linear regression first difference models of grade point averages among white females only (no association had been found for non-white females or for males). **Step 1**: adjusted for aspirations to attend university, whether had sexual intercourse, whether in a romantic relationship, parental involvement in schoolwork, parent's labor market participation, alcohol consumption, religious attendance, athletic activity, and parental setting of weekend time limits. **Step 2**: step 1 + school absence.	Step 1: a unit increase in Body Mass Index was associated with a −0.031 (95% CI −0.0604, −0.002) reduction in grade point average. Step 2: Further adjusting for absence did not affect this association (−0.033 [−0.0604, −0.0056]).	**No evidence of mediation via absence**.	Nationally representative.	All measures self-reported.
Veldwijk et al. (45) [Santana et al. (39) and Martin et al. (31)]	Obesity, 1, 543	The Netherlands (Prevention and Incidence of Asthma and Mite Allergy birth cohort)	Linear regression of standardized test z-scores. **Step 1**: unadjusted. **Step 2**: adjusted for parental education level, skipping breakfast, and screen time (other confounders excluded based on statistical significance). **Step 3**: step 2 + being bulled. **Step 4**: step 2 + mental health inventory score. **Step 5**: step 2 + health problems that affect school performance. **Step 6**: step 3 + school absence. **Step 7**: step 4 + school absence. **Step 8**: step 5 + school absence.	Step 2: After adjusting for parental education, skipping breakfast, and screen time, overweight was associated with a 0.16 reduction in test z-scores (95% CI −0.32, 0.00). Further adjustments did not significantly attenuate this coefficient (steps 3 to 5): being bullied (−0.13 [−0.29, 0.03]), mental health inventory score (−0.13 [−0.29, 0.03]), mental health problems (−0.16 [−0.32, 0.00]). Further adjusting for school absence likewise did not attenuate the associations (steps 6 to 8): being bullied and school absenteeism (−0.14 [−0.29, 0.02]), mental health inventory score and school absenteeism (−0.13 [−0.29, 0.03]) and mental health problems and school absenteeism (−0.16 [−0.32, −0.00]).	**No evidence of mediation via absence**.	Height and weight measured by a trained research assistant at age 8. Nationally representative. Health measured prior to educational outcomes.	School performance at age 12, as well as height and weight at age 12, were reported by parents. Confounders were included in models based on statistical significance rather than theory.

CHC, chronic health condition; CI, confidence interval; OR, odds ratio.

These seven studies were all affected by limitations (Table 2). Most commonly, the studies relied on self- or parent-reported measures of health or educational outcomes (or both) and so may have been affected by recall or social desirability bias in addition to selection and attrition bias inherent in longitudinal surveys. Only one study (of orofacial clefts) used measures of health and education not reported by participants or parents (44). This study instead used data from administrative health and education records linked to a national cleft registry (comparing children with cleft palate or cleft lip and palate with cleft lip only), thereby also limiting the risk of selection or attrition bias. However, this study was limited to children with orofacial clefts, and it did not include a non-symptomatic control group.

Details of other mediators hypothesized by review authors, many of which are condition-specific, are given in Table 3.

**Table 3:** Mediators hypothesized in the 27 reviews.

**Author**	**Health conditions**	**Hypothesized mediators (other than absence)**	**Hypothesized in review that absence mediated differences in attainment?**
**Generic or multiple**			
McKinley Yoder and Cantrell (1)	Generic / multiple: Chronic condition or disability	Other hypothesized mediating factors included low parental education, cognitive effects of disease due to chronic anemia and limited access to education and other resources. Epilepsy can cause brain cell death due to hypoxia, affecting a student's ability to engage in learning in addition to the effects of medication.	Yes, absence due to pain, fatigue, and crisis was hypothesized to mediate the relationship between disability and poor educational outcomes.
Hale et al. (22)	Generic / multiple: Chronic conditions	Poor health may “tax resources” resulting in insufficient time and energy devoted to school. Poor physical and mental health may result in social exclusion, which themselves are associated with lower attainment. Many mental health conditions may be associated with deficits in academic ability, behavioral difficulties, and substance use and abuse (e.g., ADHD and conduct disorders). Poor mental health may also affect skills such as verbal and other cognitive abilities. Bidirectional pathways are hypothesized.	Yes.
Moser et al. (33)	Generic / multiple: Cystic fibrosis, hemophilia A, end-stage renal disease and end-stage liver disease	The burden of hemophilia (higher number of bleeds) was posited as one cause of poorer results. End-stage renal disease and end-stage liver disease were hypothesized to affect early brain development.	Yes.
Glinianaia et al. (27)	Major structural congenital anomalies	Mediation from exposure to neurotoxic anesthetic agents resulting in neurodevelopmental and cognitive impairments was hypothesized. However, there is accumulating evidence that delayed intrauterine brain maturation, white matter injury resulting from impaired fetal hemodynamics, consequent brain immaturity at birth and longer time to surgery are the primary causes of hypoxic brain injury and subsequent poorer neurodevelopment after surgery. Psychological factors including self-confidence and self-efficacy in school were also suggested as potential mediating pathways.	Yes.
Esch et al. (2)	Mental disorders	Most psychiatric disorders present symptom patterns that can cause emotional, cognitive, and social impairment, involving a downward spiral of negative school experiences, resulting in early school leaving. Externalizing may be represented as “trouble making.” Children with reduced conceptual and procedural competencies may experience more difficulties and frustrations regarding educational success, thus engaging in externalizing behaviors whereas children with reduced social skills may adopt an internalizing coping style which is less strongly associated with school dropout. Other mediating factors may include school climate and family functioning.	No.
**Condition-specific**			
Milton et al. (19)	Asthma	Other possible causal pathways are not explicated in the paper.	Yes.
Schneider (38)	Asthma	There may be direct effects of asthma due to physical constraints affecting daily routines and overall quality of life. Compromised cognitive ability (cause unspecified by the authors), fatigue, and social distress might also contribute to a student's failure to achieve their potential.	Yes. The authors state that given absenteeism was higher among children with uncontrolled asthma or asthma of increasing severity, then addressing asthma management and control are key in bridging the achievement gap between children with and without asthma, implicitly postulating a causal link between asthma, absenteeism, and achievement.
Polderman et al. (37)	Attention problems including ADHD	Educational attainment may be affected by impaired cognitive function (e.g., regulatory control, memory, learning) or lower IQ in children with ADHD. Additionally, failure to develop basic skills in the early years because of ADHD may affect later achievement. School factors may also be important, for example the role that capable teachers have to play in creating a positive learning environment. ADHD is often comorbid with other conditions that may affect achievement, such as conduct disorder and mood and anxiety disorders.	No.
Schulte et al. (40)	Cancer: Central nervous system tumor survivors	The treatments of CNS cancers (surgery, chemotherapy, and radiation therapy) may cause structural and functional changes in the brain that adversely affect targeted and surrounding tissues and organ systems.	No.
Langevald et al. (29)	Cancer: Survivors of childhood cancer	Treatments may cause debilitating deficits.	No.
Molcho et al. (32)	Cancer: Survivors of childhood cancer	Due to toxicity of treatment, survivors may experience adverse late effects, including physical, social, and emotional problems. Alongside pain, emotional problems, and impaired mental health, survivors may experience neurocognitive dysfunction affecting educational outcomes. Exposure to cranial radiation may explain why children with CNS tumors have lower attainment. Studies that found no difference or favorable outcomes among survivors might be explainable based on extra provision made for these children or differences in treatments (not reported in the studies).	Yes, particularly absence due to treatment. Absence has been shown to be highest immediately following treatment initiation but irregular school attendance may persist for years.
Saatci et al. (36)	Cancer: Survivors of childhood cancer	Particularly in the case of central nervous system tumors, brain involvement, and resultant cognitive functioning may explain lower attainment. Not taking measures to ensure successful school re-entry may also be a factor.	No.
Chen et al. (24)	Chronic kidney disease	Metabolic, biochemical, and neurodegenerative mechanisms (e.g., through neurotoxic demyelination caused by increased plasma levels of uremic solutes) may lead to lower intellectual function. Treatment may also affect academic performance, e.g., through sleep disturbance leading to day-time impaired concentration or side effects of medication.	Yes, absenteeism due to on-going dialysis sessions and recovery from transplant surgeries is hypothesized to result in potential loss of interest, withdrawal from school, and poor school progression.
Alsaggaf and Coyne (23)	Chronic pain	In qualitative studies, it was stated that academic difficulties stemmed from academic competence, difficulties with concentration, time and effort getting schoolwork done, and comprehension and memory. Accommodations and collaboration with parents discussed as important in improving school function. Lack of knowledge of school personnel on how to manage chronic pain or its biopsychosocial nature were cited as barriers. Review authors conclude, based on conflicting findings as to academic achievement, that success may be impaired when pain has an impact on cognition whereas when support is received, the young person performs better.	Yes. One study reported that 68% of experienced teachers and 58% of student teachers perceived attendance to be an obstacle to academic success.
Ragnarsson et al. (35)	Chronic pain	Recurrent pain results in poorer sleep which may cause tiredness and hamper ability to keep up with schoolwork. Children with chronic pain are also prone to concentration problems, impaired executive function and impaired school functioning. These in turn may affect achievement/attainment at school. The association may be bidirectional with difficulties at school exacerbating pain problems though this was only tested in one study in the review (no association between achievement and pain observed).	Yes, and the authors state that school success among children with recurrent pain may be enhanced by minimizing school absenteeism and providing homework support.
Cocomello et al. (26)	Congenital heart disease	Exposure to neurotoxic factors affecting brain development, such as cyanosis and neurotoxicity related to cardiopulmonary bypass and hypothermic circulatory arrest in children undergoing heart surgery. Chromosomal abnormality, such as Down's syndrome, is often associated with CHD and may contribute to lower attainment. Incidence of psychological and psychiatric disorders has been reported to be higher in patients with congenital heart disease and these may affect academic performance.	Yes, particularly absence due to recurrent chest infection, endocarditis, cardiac arrhythmias, or repeated surgeries.
Clayborne et al. (25)	Depression	Depression is associated with functional impairment, which may have negative effects on comprehension and ability to complete schoolwork. Adolescents with depression may leave school earlier due to disinterest, functional impairment and/or truancy. Although the present study attempted to rule out reverse causation by including studies where depression was measured at least 12 months before the outcome, reverse causality between poor attainment and depression cannot be ruled out.	Yes, depression hypothesized to affect attendance and, therefore, attainment and peer relationships.
Wickersham et al. (42)	Depression	The pathway between depression and attainment potentially comprises a wide range of pupil-, parent-, teacher- and school-level factors. Reduced energy, motivation, and concentration may affect engagement, attendance, and performance at school. From mediation analyses of the included studies, the authors further hypothesized that lack of social support may also be a mechanism. Other possible factors include socioeconomic position, relative age in year, teacher support, pupil engagement, school involvement and academic self-sufficiency. The association may be bi-directional.	Yes.
Puka et al. (43)	Epilepsy	Neurological, cognitive and psychiatric problems are cited as potential causes of poorer outcomes in people with epilepsy. However poor outcomes have also been found in people with epilepsy who do not have such comorbidities. Targets for further research include person- and environment-level factors and their interactions. Disease-related factors such as seizure frequency, anti-epileptic drug use, whether seizures are controlled may be relevant as may access to transportation or driving, family environment, mental health problems, extent of perceived and/or enacted stigma and external vs internal locus of control.	No.
Wo et al. (20)	Epilepsy	Children with epilepsy may, due to the disease itself or its treatment, have specific learning problems such as inattention and working memory that influence classroom learning and academic achievement. Psychomotor impairment and impairment of affective domains might also affect learning. Family factors may also contribute to academic difficulties, as may parental mental health, including anxiety, problems. The child's attitude to their illness, self-esteem and motivation have also been cited as potential causes as have teachers' involvement.	No.
Lah et al. (28)	Epilepsy (temporal lobe)	Seizure focus is often not restricted to the hippocampus but also the temporal neocortex, an integral part of the reading network. Seizures can interfere with knowledge and skills acquisition. Neurocognitive deficits could contribute to reading difficulties, such as deficits with episodic or semantic memory, learning and recall.	Yes.
Caird et al. (17)	Obesity	Other potential mechanisms identified included mediation through poor mental health, discrimination/stigmatization, sleep, cardiovascular risk (mechanism unspecified), and micronutrient deficiency leading to lower cognitive ability. Some studies also hypothesized that obesity may lead to decreased physical activity and socializing, which in turn lead to more time studying and hence higher attainment.	Yes. A previous literature review suggested that, inter alia, increased absenteeism may explain an observed correlation between obesity and lower academic performance. One study included in this review explicitly posited school absence as a mediator but did not test this pathway.
Martin et al. (31)	Obesity	Mechanisms proposed were: “direct” mechanisms through cognitive ability and “indirect” mechanisms including obesity-related psychological distress, depression and internalizing behavior, stigmatization, self-efficacy, age at menarche, poor sleep due to obesity-related disordered breathing, cardio-metabolic comorbidities, nutritional intake and low levels of physical activity or fitness. Weight-related bullying of girls, in particular, may lead to lower academic achievement. In focus groups conducted by the review authors, adolescent girls stated that they generally have a positive attitude to school and often outperform healthy-weight peers except in physical education. Respondents reported that they may have better grades due to lack of friends and absence of good peer relationships.	Yes. The authors hypothesize that obesity-related adverse mental and physical health may lead to increased school absenteeism and lower achievement.
Santana et al. (39)	Obesity	Some studies have shown that obesity is associated with lower IQ and that those with obesity exhibit poorer executive function, memory, attention and motor skills. Adiposity may directly affect cognition. Alternatively, weight-related bias and discrimination may influence self-esteem by internalizing and externalizing behavior problems. Anxiety/depression may also play a mediating role in the obesity-academic performance relationship.	No.
Segal et al. (41)	Obesity	The authors propose that adolescence may be a critical period for obesity to produce negative effects or that the detrimental effects of obesity may accumulate across childhood, explaining the findings that obesity was most consistently associated with poorer attainment at older ages. However, they do not propose specific pathways.	No.
Milton et al. (30)	Type 1 diabetes	Poor glycemic control may lead to episodes of hypoglycaemia affecting the developing nervous system, especially in younger children. Adolescents may exhibit deteriorating metabolic control.	Yes, stress, poor metabolic control and diabetic complications may affect school attendance, in turn affecting performance.
Oakley et al. (34)	Type 1 diabetes	Diabetes complications include hypo- and hyperglycaemia and ketoacidosis, which especially if recurrent, have the potential to impact educational attainment via altered cognitive function.	Yes, attendance may be affected by the need for acute treatment, in turn affecting attainment.

## 4. Discussion

We found evidence of strong associations between CHCs and educational attainment but a lack of evidence that this association is mediated by school absence. Our umbrella review of 27 systematic reviews (1, 2, 17, 19, 20, 22–43), covering 441 unique research studies of 7.5 million participants from 47 regions, found evidence that children with CHCs generally, or with major structural congenital anomalies, mental disorders, attention problems, central nervous system tumors, chronic kidney disease, chronic pain, congenital heart disease, and depression, were more likely than peers without the relevant CHCs to have lower academic attainment. Evidence for children with asthma, epilepsy, and obesity was mixed with studies finding either very weak associations with lower academic attainment, no associations, or even associations with higher academic attainment.

Only 7 of 441 studies (2%) empirically tested the hypothesis that absence from school is a mediator in this relationship (44–50). Of these seven, which included analyses of asthma, obesity, orofacial clefts, and cancer/diabetes/epilepsy (as one variable), none found any evidence that absence was a mediator. We, therefore, conclude that whereas there is strong evidence that a range of CHCs is associated with lower academic attainment, the hypothesized mediating pathway between CHCs, school absence, and academic attainment (Figure 1) currently has no strong empirical foundation in either direction.

The strengths of our umbrella review are the broad search and inclusion of a large number of studies. Although many of the reviews only included English-language studies, our finding that only 2% of 441 studies explored the extent to which absence mediates the association between CHCs and academic attainment shows a clear gap in the evidence about the mechanism through which CHCs might lead to lower attainment. A limitation is the varied types of comparator groups and varied adjustments for confounding factors. The wide range of conditions and study designs meant that meta-analysis was inappropriate; however, qualitatively, there were consistent findings on the associations between a range of CHCs (*vs*. none or different levels of CHC severity) and academic attainment. Additionally, our review did not aim to examine whether other possible mediators (such as those documented in Table 3) do in fact mediate the association between CHCs and academic attainment. Given the heterogeneous nature of the conditions under study, future CHC-specific studies will be required to further elucidate these factors.

Intervening to improve the educational outcomes of children with CHCs requires understanding the root causes of absence in these children, which likely differ between different CHCs and among children without CHCs. CHCs are very common, affecting up to 27% of young people in early adolescence (14). In England, absence from school is also common and, of all absences, the majority (73% in 2018/19) are authorized and, of these, 63% are due to either illness or needing to attend healthcare services (51). Among children with CHCs, the root causes of absence may relate to the condition itself, its management, or the need to attend healthcare appointments. There may be a common cause, for example, times of acute illness may prevent school attendance while also undermining cognitive function. School attendance and absence policies should therefore view absence as a potential health issue and respond flexibly, in accordance with equalities legislation, and provide sufficient resources to enable the affected young person to stay engaged with education both in and out of school.

The findings from this review have implications for policy and research. First, policies that solely target reductions in absence might not improve attainment and could be harmful to children with CHCs. However, operating different policies for children with and without CHCs would require asking questions of children and their parents about their health conditions. This could be experienced as intrusive and stigmatizing and undermine relationships with school staff. Identification of CHCs could also drive demand for unnecessary health investigations or evidence from medical staff, which could breach patient confidentiality. The implication for policy is that any efforts that address the common causes, whether rooted in health or social needs, may be more effective for increasing participation in school, in turn improving attainment and wellbeing, and avoid alienation and stress, particularly for children with CHCs.

More research is needed to identify potential interventions to support participation in education and attainment of children with CHCs. Studies using administrative data can help to plug the current evidence gap (52), including comparisons between jurisdictions with different approaches in schools and/or healthcare. There is an urgent need for randomized-controlled trials of interventions, developed with the input of children and young people and their families, within education and/or healthcare services, to identify approaches that promote child wellbeing and improve participation in education and attainment among children with CHCs.

## Data availability statement

The original contributions presented in the study are included in the article/Supplementary material, further inquiries can be directed to the corresponding author.

## Author contributions

MJ designed the study to which all authors contributed and deduplicated the articles and carried out initial synthesis and article drafting to which all the authors contributed. MJ and DS-E carried out article eligibility assessment and data extraction as documented in the methods section of this manuscript. All the authors contributed to the manuscript revision and read and approved the submitted version.
