# Supplementary material for: Umbrella systematic review finds limited evidence that school absence explains the association between chronic health conditions and lower academic attainment
Source: Front Public Health. 2023 Jun 9;11:1122769. doi: 10.3389/fpubh.2023.1122769 (PMC10288991; doi:10.3389/fpubh.2023.1122769)
Supplement: Supplementary file 2 [file Table_2.DOCX]

**Supplementary File 2 – search strategy**

Supplementary Table 2.1. Search strategy used to identify systematic reviews on chronic health conditions and academic attainment in Medline, Embase and PsycINFO (Ovid): 27 September 2021.

| Key word | Number of hits |
| --- | --- |
|  |  |
| *Health keywords* |  |
| 1. (chronic condition* or chronic disease* or congenital anomal* or inborn error or chronic health condition* or chronic or asthma or wheeze or cleft lip or cleft palate or cleft lip and palate or autism or ASD or autis* spectrum disorder* or neuro* disorder or neuro* disability or chronic pain or intractable pain or recurrent pain or cancer or malignan* or diabetes or depression or anxiety or mental health dis* or mental health condition* or obes* or chronic fatigue or myalgic encephalomyelitis or “CFS/ME” or cystic fibrosis or renal disease or kidney disease or heart disease or lung disease or liver disease or epilepsy or epileptic* or seizure* or sickle cell).tw | 10,397,234 |
|  |  |
| *Education keywords (adapted from Caird et al 2013)* |  |
| 2. (GCSE or GCSEs or SATs or Assessment tests or school diploma or educational development or school certificate* or national tests or national testing or secondary education or primary education or national examinations or Key Stage or grade scores or ordinary level or O-level* or academic test* or academic examination or academic exam or academically attain* or academic measure* or academic assess* or academic evaluat* or academic standard* or academic deficien* or academically deficien* or academically perform or academic performance* or academic achievement* or academic outcome* or academic failure* or academic level* or school perform* or school standard* or school evaluat* or school assess* or school test* or school outcomes or school examination or school exam or school measure* or school performance* or school achievement* or school failure* or school exams or test scores or testing scores or education test* or educational attain* or educational measure* or education assess* or education evaluat* or education standard* or educational standard* or education deficien* or educational deficien* or educationally deficien* or education perform or education performance* or education achievement* or educational achievement* or education level* or educational level* or education failure* or education outcome* or education exams or educational failure* or educational outcome* or educational test* or examination perform* or examination standard* or examination assess* or examination outcomes or examination performance* or examination achievement* or examination failure* or engagement or attainment*).tw | 594,900 |
|  |  |
| *Systematic reviews* |  |
| 3. systematic review.ti | 337,793 |
|  |  |
| *Combine, limit and deduplicate* |  |
| 4. 1 and 2 and 3 | 1,308 |
| 5. Limit 4 to "all child (0 to 18 years)" [Limit not valid in Embase,APA PsycInfo; records were retained] | 1,034 |
| 6. Limit 5 to (childhood <birth to 12 years> or adolescence <13 to 17 years>) [Limit not valid in Embase,Ovid MEDLINE(R); records were retained] | 834 |
| 7. Limit 6 to child <unspecified age> [Limit not valid in Ovid MEDLINE(R),APA PsycInfo; records were retained] | 287 |
| 8. Deduplicate 7 | 205 |
|  |  |

.tw = title and abstract. .ti = title only.

Supplementary Table 2.2. Search strategy used to identify systematic reviews on chronic health conditions and academic attainment in the Education Resources Information Center and the Education Database (ProQuest): 14 October 2021

| Key word | Number of hits |
| --- | --- |
|  |  |
| *Health keywords* |  |
| (ti(chronic condition* or chronic disease* or congenital anomal* or inborn error or chronic health condition* or chronic or asthma or wheeze or cleft lip or cleft palate or cleft lip and palate or autism or ASD or autis* spectrum disorder* or neuro* disorder or neuro* disability or chronic pain or intractable pain or recurrent pain or cancer or malignan* or diabetes or depression or anxiety or mental health dis* or mental health condition* or obes* or chronic fatigue or myalgic encephalomyelitis or “CFS/ME” or cystic fibrosis or renal disease or kidney disease or heart disease or lung disease or liver disease or epilepsy or epileptic* or seizure* or sickle cell) OR ab(chronic condition* or chronic disease* or congenital anomal* or inborn error or chronic health condition* or chronic or asthma or wheeze or cleft lip or cleft palate or cleft lip and palate or autism or ASD or autis* spectrum disorder* or neuro* disorder or neuro* disability or chronic pain or intractable pain or recurrent pain or cancer or malignan* or diabetes or depression or anxiety or mental health dis* or mental health condition* or obes* or chronic fatigue or myalgic encephalomyelitis or “CFS/ME” or cystic fibrosis or renal disease or kidney disease or heart disease or lung disease or liver disease or epilepsy or epileptic* or seizure* or sickle cell)) | 171,786 |
|  |  |
| *Education keywords (adapted from Caird et al 2013)* |  |
| (ti(GCSE or GCSEs or SATs or Assessment tests or school diploma or educational development or school certificate* or national tests or national testing or secondary education or primary education or national examinations or Key Stage or grade scores or ordinary level or O-level* or academic test* or academic examination or academic exam or academically attain* or academic measure* or academic assess* or academic evaluat* or academic standard* or academic deficien* or academically deficien* or academically perform or academic performance* or academic achievement* or academic outcome* or academic failure* or academic level* or school perform* or school standard* or school evaluat* or school assess* or school test* or school outcomes or school examination or school exam or school measure* or school performance* or school achievement* or school failure* or school exams or test scores or testing scores or education test* or educational attain* or educational measure* or education assess* or education evaluat* or education standard* or educational standard* or education deficien* or educational deficien* or educationally deficien* or education perform or education performance* or education achievement* or educational achievement* or education level* or educational level* or education failure* or education outcome* or education exams or educational failure* or educational outcome* or educational test* or examination perform* or examination standard* or examination assess* or examination outcomes or examination performance* or examination achievement* or examination failure* or engagement or attainment*) or ab(GCSE or GCSEs or SATs or Assessment tests or school diploma or educational development or school certificate* or national tests or national testing or secondary education or primary education or national examinations or Key Stage or grade scores or ordinary level or O-level* or academic test* or academic examination or academic exam or academically attain* or academic measure* or academic assess* or academic evaluat* or academic standard* or academic deficien* or academically deficien* or academically perform or academic performance* or academic achievement* or academic outcome* or academic failure* or academic level* or school perform* or school standard* or school evaluat* or school assess* or school test* or school outcomes or school examination or school exam or school measure* or school performance* or school achievement* or school failure* or school exams or test scores or testing scores or education test* or educational attain* or educational measure* or education assess* or education evaluat* or education standard* or educational standard* or education deficien* or educational deficien* or educationally deficien* or education perform or education performance* or education achievement* or educational achievement* or education level* or educational level* or education failure* or education outcome* or education exams or educational failure* or educational outcome* or educational test* or examination perform* or examination standard* or examination assess* or examination outcomes or examination performance* or examination achievement* or examination failure* or engagement or attainment*)) | 981,443 |
|  |  |
| *Systematic reviews* |  |
| (ti(“systematic review”)) | 4,830 |
|  |  |
| *Combine, limit and deduplicate* |  |
| All three above terms joined by AND | 311 |
| Limit to children, adolescents, teenagers or children & youth | 107 |
| Deduplicate in Mendeley | 95 |
|  |  |

ti = sarch in title. ab = search in abstract.

Supplementary Table 2.3. Search strategy used to identify systematic reviews on gastro chronic conditions and academic attainment in Medline, Embase and PsycINFO (Ovid): 22 March 2022.

| Key word | Number of hits |
| --- | --- |
|  |  |
| *Health keywords* |  |
| 1. (gastro* or IBD or "irritable bowel" or Chron’s or Colitis or digestive).tw | 1,307,053 |
|  |  |
| *Education keywords (adapted from Caird et al 2013)* |  |
| 2. (GCSE or GCSEs or SATs or Assessment tests or school diploma or educational development or school certificate* or national tests or national testing or secondary education or primary education or national examinations or Key Stage or grade scores or ordinary level or O-level* or academic test* or academic examination or academic exam or academically attain* or academic measure* or academic assess* or academic evaluat* or academic standard* or academic deficien* or academically deficien* or academically perform or academic performance* or academic achievement* or academic outcome* or academic failure* or academic level* or school perform* or school standard* or school evaluat* or school assess* or school test* or school outcomes or school examination or school exam or school measure* or school performance* or school achievement* or school failure* or school exams or test scores or testing scores or education test* or educational attain* or educational measure* or education assess* or education evaluat* or education standard* or educational standard* or education deficien* or educational deficien* or educationally deficien* or education perform or education performance* or education achievement* or educational achievement* or education level* or educational level* or education failure* or education outcome* or education exams or educational failure* or educational outcome* or educational test* or examination perform* or examination standard* or examination assess* or examination outcomes or examination performance* or examination achievement* or examination failure* or engagement or attainment*).tw | 622,496 |
|  |  |
| *Systematic reviews* |  |
| 3. systematic review.ti | 373,946 |
|  |  |
| *Combine, limit and deduplicate* |  |
| 4. 1 and 2 and 3 | 31 |
| 5. Limit 4 to "all child (0 to 18 years)" [Limit not valid in Embase,APA PsycInfo; records were retained] | 28 |
| 6. Limit 5 to (childhood <birth to 12 years> or adolescence <13 to 17 years>) [Limit not valid in Embase,Ovid MEDLINE(R); records were retained] | 25 |
| 7. Limit 6 to child <unspecified age> [Limit not valid in Ovid MEDLINE(R),APA PsycInfo; records were retained] | 9 |
| 8. Deduplicate 7 | 9 (no duplicates) |
|  |  |

.tw = title and abstract. .ti = title only.

Supplementary Table 2.4. Search strategy used to identify systematic reviews on gastro chronic conditions and academic attainment in the Education Resources Information Center and the Education Database (ProQuest): 22 March 2022

| Key word | Number of hits |
| --- | --- |
|  |  |
| *Health keywords* |  |
| (ti(gastro* or IBD or "irritable bowel" or Chron* or Colitis or digestive) OR ab(gastro* or IBD or "irritable bowel" or Chron’s or Colitis or digestive)) | 2,028 |
|  |  |
| *Education keywords (adapted from Caird et al 2013)* |  |
| (ti(GCSE or GCSEs or SATs or Assessment tests or school diploma or educational development or school certificate* or national tests or national testing or secondary education or primary education or national examinations or Key Stage or grade scores or ordinary level or O-level* or academic test* or academic examination or academic exam or academically attain* or academic measure* or academic assess* or academic evaluat* or academic standard* or academic deficien* or academically deficien* or academically perform or academic performance* or academic achievement* or academic outcome* or academic failure* or academic level* or school perform* or school standard* or school evaluat* or school assess* or school test* or school outcomes or school examination or school exam or school measure* or school performance* or school achievement* or school failure* or school exams or test scores or testing scores or education test* or educational attain* or educational measure* or education assess* or education evaluat* or education standard* or educational standard* or education deficien* or educational deficien* or educationally deficien* or education perform or education performance* or education achievement* or educational achievement* or education level* or educational level* or education failure* or education outcome* or education exams or educational failure* or educational outcome* or educational test* or examination perform* or examination standard* or examination assess* or examination outcomes or examination performance* or examination achievement* or examination failure* or engagement or attainment*) or ab(GCSE or GCSEs or SATs or Assessment tests or school diploma or educational development or school certificate* or national tests or national testing or secondary education or primary education or national examinations or Key Stage or grade scores or ordinary level or O-level* or academic test* or academic examination or academic exam or academically attain* or academic measure* or academic assess* or academic evaluat* or academic standard* or academic deficien* or academically deficien* or academically perform or academic performance* or academic achievement* or academic outcome* or academic failure* or academic level* or school perform* or school standard* or school evaluat* or school assess* or school test* or school outcomes or school examination or school exam or school measure* or school performance* or school achievement* or school failure* or school exams or test scores or testing scores or education test* or educational attain* or educational measure* or education assess* or education evaluat* or education standard* or educational standard* or education deficien* or educational deficien* or educationally deficien* or education perform or education performance* or education achievement* or educational achievement* or education level* or educational level* or education failure* or education outcome* or education exams or educational failure* or educational outcome* or educational test* or examination perform* or examination standard* or examination assess* or examination outcomes or examination performance* or examination achievement* or examination failure* or engagement or attainment*)) | 1,001,347 |
|  |  |
| *Systematic reviews* |  |
| (ti(“systematic review”)) | 5,245 |
|  |  |
| *Combine, limit and deduplicate* |  |
| All three above terms joined by AND | 3 |
| Limit to children | 2 |
| Deduplicate in Mendeley | 2 (no duplicates) |
|  |  |

ti = sarch in title. ab = search in abstract.
