# Supplementary material for: Umbrella systematic review finds limited evidence that school absence explains the association between chronic health conditions and lower academic attainment
Source: Front Public Health. 2023 Jun 9;11:1122769. doi: 10.3389/fpubh.2023.1122769 (PMC10288991; doi:10.3389/fpubh.2023.1122769)
Supplement: Supplementary file 3 [file Table_3.docx]

**Supplementary File 3 – review details**

Supplementary Table 3.1. Summary of 27 included systematic reviews

| Author | Health conditions | Outcomes | Language | Years | Study inclusion | Number of studies on academic attainment / total number of studies included | Countries of studies on academic attainment | Min to max sample size (median) of studies on academic attainment | Comparison groups of studies on education academic attainment | Results on academic attainment |
| --- | --- | --- | --- | --- | --- | --- | --- | --- | --- | --- |
| **Generic or multiple** | | | | | | | | | | |
| McKinley Yoder & Cantrell 2019 | Generic / multiple: Chronic condition or disability | Grade retention, high school graduation and school absence | English | 1990 to 2018 | Quantitative or mixed-methods designs of school-aged children (5-21 years) in a peer-reviewed journal with a research population in the USA. | 26/42 examining high school graduation and/or grade repetition | USA (26) | 36 to 59,440 (2,247) | Comparisons were with children without chronic health conditions/ disability | Studies using national data examined high school completion and found that, for a range of conditions, having a chronic health condition was associated with lower chance of completing high school. This may include significant effects of early health conditions on later outcomes: in one study (Wisk & Weitzman, 2017), having a condition diagnosed before age 14 was associated with not completing high school after age 14. Studies generally adjusted for socioeconomic position and race/ethnicity. In small studies, grade repetition was associated with epilepsy, liver disease and kidney disease. |
| Hale et al 2015 | Generic / multiple: Chronic conditions | Education level by adulthood (including completion of secondary education) | English | 1990 to study | Longitudinal studies with at least two years between health and outcome measurements. Studies must have had comparison groups of health controls. | 14/27 included in meta-analysis of not completing secondary education and a further 1/27, not included in meta-analysis, examining 9 or fewer years of education. | USA (8), New Zealand (2), Canada (1), China (1), France (1), Sweden (1) | 91 to 14,221 (317) | Healthy controls | Overall, those with any mental or physical health condition (depression, ADHD, conduct disorder, psychiatric disorder, JIA, "any chronic condition"), those with a condition were more likely to not complete secondary education than those with (OR [95% CI] 2.07 [1.50, 2.86]). However, in analyses disaggregated by condition type, only mental health conditions (14 studies) were associated with not completing secondary education (2.43 [1.68, 3.53); from the two studies of physical conditions, there was no association (0.98 [0.43, 2.27]). The one study examining whether participants had 9 or fewer years of education found no association between this outcome and anorexia nervosa. |
|  |  |  |  |  |  |  |  |  |  |  |
| Moser et al 2013 | Generic / multiple: Cystic fibrosis, haemophilia A, end-stage renal disease and end-stage liver disease | Neurocognitive outcomes measured using standardised achievement tests | English | Database inception to 2012 | Studies of children aged 1-18 with one of the four conditions that reported results for achievement tests. | 9/21 (the remainder examined IQ or factors such as cognitive development) | USA (8), Canada (1) | 22 to 333 (40) | Population norms | One study on CF found no differences in attainment. Five studies on haemophilia A found lower performance on tests compared to population norms. One study of ESRD found that children post transplant performed better in written language and school performance than dialysed peers. Of the two studies of ESLD, both showed transplant recipients performed worse academically. |
|  |  |  |  |  |  |  |  |  |  |  |
| Glinianaia et al 2021 | Generic / multiple: Major structural congenital anomalies | Academic achievement or receipt of special educational needs provision | English | 1990 to 2020 | Any design examining academic performance or special educational needs in children with a congenital anomaly vs a comparison group or normative data. | 32/39 | USA (17), UK (5), Australia (4), Denmark (2), Norway (1), USA & Canada (1), Switzerland (1), Sweden (1) | 12 to 1,251,396 (199) | Studies had to have a control group or compare with normative data | Most studies found that children with spina bifida, CHD or severe CHD, or orofacial clefts (especially with palate involvement) had lower average attainment. These findings remain persistent across school levels. |
|  |  |  |  |  |  |  |  |  |  |  |
| Esch et al 2014 | Generic / multiple: Mental disorders | School dropout | English, French or German | 1990 (to not include references before DSM-III, though this was published in 1980) to 2014 | Cross-sectional, case-control, longitudinal or meta-analyses were included. School dropout in secondary school prior to graduation. Papers could examine the association between mental disorders and school dropout in either direction. Mental health disorders (apart from substance use) had to refer to the DSM. | 34/51 | USA (21), Australia (3), Canada (3), New Zealand (2), Australia and New Zealand (1), Sweden (1),  World Mental Health Survey (1), France (1), Mexico (1) | 36 to 41,688 (1,329). | Comparison groups were not pre-specified but studies had to examine an association between a mental disorder and school dropout. A small number of studies used clinical samples. | Substance use and use disorders (cannabis [with the exception of experimentation without progression to daily use], nicotine or amphetamines), externalising disorders, ADHD and psychosis were most strongly associated with school dropout. Internalising disorders were also associated with an increased likelihood of school dropout but less strongly. Where studies made adjustments for sociodemographic factors, associations remained. |
|  |  |  |  |  |  |  |  |  |  |  |
| **Condition-specific** | | | | | | | | | | |
| Milton et al 2004 | Asthma | Academic attainment and school absence | English | 1975 to 2002 | Industrialised countries with participants aged less than 18 at time of asthma onset. | 12/29 | USA (7), UK (3), Netherlands (1), New Zealand (1) | 50 to 13,509 (273) | Asymptomatic controls or general population data | Absence rates were higher among children with asthma compared to those without, however, there were no differences in attainment except in one small study (n=50) which found severe asthma was associated with lower reading and mathematics scores at baseline. The majority of research was cross-sectional or case-control. |
|  |  |  |  |  |  |  |  |  |  |  |
| Schneider 2020 | Asthma | Educational achievement | English (studies in North America only) | 2000 to 2017 | 10-18 year olds with an outcome pertaining to academic achievement. | 10/20 | USA (9), Canada (1) | 57 to 300,881 (5706) | Healthy controls | Out of eight observational studies, 5 showed that students with asthma performed worse academically than controls. Of two RCTs, both showed improvements in grades following treatment. |
|  |  |  |  |  |  |  |  |  |  |  |
| Polderman et al 2010 | Attention problems including ADHD | Academic achievement (including grade retention, drop-out, years of schooling and standardised tests) | No limits | Database inception to 2009 | Longitudinal studies of attention deficit, hyperactivity/impulsivity or attention problems with academic achievement as outcome measures and no medical manipulation or focus on comorbid conditions. | 15/16 (one study focused on effort in school work and confidence in performance) | USA (11), UK (2), USA, UK & Canada (1), USA & Canada (1) | 73 to 34,837 (301) | Analyses were either case vs control or were of continuous measures in "normal" populations | There was strong evidence (i.e., from two studies with the lowest risk of bias) that inattentive symptoms were predictive of lower academic achievement. In these two studies, hyperactive symptoms were no longer associated with achievement after adjusting for IQ and SES. There was, however, evidence from other studies at higher risk of bias that hyperactive symptoms were also a predictor of lower achievements. |
| Schulte et al 2019 | Cancer: Central nervous system tumour survivors | Social attainment (including not graduating/completing compulsory education) | English | 2011 to 2018 | Diagnosed with CNS tumour <21 yrs of age and describing outcomes at least 5 years post-diagnosis or 2 years post-treatment. | 4/45 included in meta-analysis | Switzerland (1), Sweden (1), Finland (1), Norway (1) | 1,523 to 121,530 (5,085) | Healthy controls or population norms (controls only in meta-analysis) | Survivors of CNS cancers were more likely to have not graduated or only complete compulsory schooling: pooled OR: 1.87 (95% CI 1.66, 2.12). |
|  |  |  |  |  |  |  |  |  |  |  |
| Langevald et al 2002 | Cancer: Survivors of childhood cancer | Quality of life (various dimensions including education) | English | Database inception to 2001 | Studies with a matched control group or comparison with measurement norms. Participants must have had at least 5 years' survival from completion of therapy. | 13/30 | USA (7), Austria (1), Finland (1), Israel (1), Netherlands (1), Norway (1), UK (1) | 40 to 5,544 (184) | Matched controls or population norms | Most studies (all retrospective self-reports) found no differences in educational attainment with the exception of two studies on CNS tumours and two on ALL, which showed lower levels of education/poorer performance in survivors compared to controls (though in one study of ALL, this only applied when the cancer was diagnosed before 7 years). |
|  |  |  |  |  |  |  |  |  |  |  |
| Molcho et al 2019 | Cancer: Survivors of childhood cancer | Academic attainment | English | 2005 to 2018 | Studies of child onset cancer (to age 18) and educational attainment (graduation of primary, secondary or tertiary education). High income countries. | 14/14 | Sweden (3), Germany (2), Netherlands (2), USA (2), France (1), Norway (1), Scandinavia (1), Switzerland (1), Turkey (1) | 201 to 1,459,262 (201) | Non-cancer control group or external population comparison | Overall, four studies reported higher attainment in cancer survivors; six reported no differences; four reported lower attainment. Only two studies were rated as high quality and ten were considered adequate. Mixed findings may therefore be a result of too few high-quality studies. Four studies of CNS tumours consistently found poorer attainment in survivors. Results for haematological cancers were mixed. |
| Saatci et al 2020 | Cancer: Survivors of childhood cancer | Educational attainment | No limits | Database inception to 2018 | Survivors of childhood cancer (remission at least 2 years) after diagnosis, comparative study, reporting an education attainment outcome (completion of compulsory schooling; non-mandatory education; higher education) | 12 examining compulsory schooling out of 26 | UK (2), USA (2), Sweden (1), Switzerland (1), Italy (1), USA & Canada (1), Netherlands (1), Finland (1), Norway (1) | to 74 to 1,457,805 (982) | Matched controls, siblings or population controls | The pooled OR for only completing secondary education by survivors of childhood cancer vs controls was 1.36 (1.28, 1.43). When examining only CNS tumour survivors, the pooled OR was higher: 1.77 (1.46 to 2.15). When examining survivors with CNS-mediated therapy (e.g., CNS tumour and leukaemia), the pooled OR was similar to the main analysis: 1.38 (1.29, 1.48). However, even among survivors with no CNS involvement, odds of completing compulsory education only remained higher: pooled OR 1.19 (1.00, 1.42). |
|  |  |  |  |  |  |  |  |  |  |  |
| Chen et al 2018 | Chronic kidney disease | Neurocognitive or academic outcomes | No limits | Database inception to 2016 | Prospective cohorts and cross-sectional designs in children and adolescents (aged <21 yr). | 10/34 | USA (8), Canada (2) | 21 to 368 (41) | Either healthy controls or between different stages (e.g., on dialysis vs received transplant) or with population norms. | Children with chronic kidney disease performed worse than those without, especially those on dialysis, at clinically meaningful levels. |
|  |  |  |  |  |  |  |  |  |  |  |
| Alsaggaf & Coyne 2020 | Chronic pain | School functioning (attendance, attainment, competence, physical activities, social relationships) and school personnel responses to managing pain in schools. | English | Database inception to 2018 | Studies were either of adolescents aged 12-19 years "medically diagnosed" with chronic pain or parents of adolescents "struggling with chronic pain" or school staff. All quantitative or qualitative designs were eligible. | 2/14 | USA (1), UK (1) | 10 and 220 | None | In one study (USA, n=220), parents reported grades before and after pain onset and parents and adolescents gave subjective reports of the impact of pain on academic performance using a 10cm visual analogue scale. Half of parents reported no change in grades and 44% reported a decline. Adolescents and parents reported moderate pain interference with performance. In the other (UK, n=10), 4/10 participants reported having repeated a school year. |
|  |  |  |  |  |  |  |  |  |  |  |
| Ragnarsson et al 2020 | Chronic pain | Academic achievement | English or Swedish | 1990 to 2018 | Recurrent pain (pain of at least 3 months' duration) and educational achievement. Studies must have had a recurrent pain-free control group. | 21/21 | USA (4), Malaysia (2), Brazil (2), Turkey (2), Finland & Sweden (1), New Zealand (1), Finland (1), Switzerland (1), Sri Lanka (1), Canada (1), Belgium (1), Greece (1), USA & Canada (1), Italy (1), Kuwait (1) | 57 to 21,065 (971) | A control group without recurrent pain | In the majority of studies, recurrent pain (studies examined headache/migraine, back pain, abdominal pain and/or musculoskeletal pain) was associated with poorer achievement, objectively measured variously as grade point average, standardised test scores or failing to obtain graduation certificates from elementary school. Effect sizes were generally small (odds ratios <2 or Cohen's d <0.5) and study quality was assessed by the authors as being low or very low overall. |
|  |  |  |  |  |  |  |  |  |  |  |
| Cocomello et al 2021 | Congenital heart disease | Educational outcomes by adulthood (including completing secondary education) | No limits | None | For the first aim of assessing the association between CHD and attainment, any study with a comparator group of non-CHD patients from the same population as patients with CHD was included. For a second aim of estimating the proportions of patients with CHD who subsequently achieve certain educational levels, studies also included if there was control group and comparisons were made with population norms. Study must have reported the rate, odds or % of educational level even if this was not the study's primary aim. | 31 with data on secondary education (+/- comparison group) and 10 with data on secondary education and a comparison group included in meta-analysis. Out of a total of 42 studies. | USA (7), Denmark (4), Switzerland (2), Germany (2), Netherlands (2), France (2), Sweden (2), Norway (1), Finland (1), South Korea (1), Belgium (1), Spain (1), International (high income) (1), Chile (1), Turkey (1), Iran (1), Malta (1) | Of the 31: 25 to 75,824 (252). Of the 10 included in meta-analysis: 248 to 68,805 (4,363). | 10 studies in the meta-analysis had a comparison group of non-CHD patients drawn from the same population as CHD patients | The weighted proportion of patients completing secondary education across all 32 studies was 84% (95% CI 76%, 90%). This compares to population norms of 70% to 91% depending on country. In pooled meta-analysis, the odds of not completing secondary education in patients with CHD vs those without were 1.33 times higher (95% CI 1.09, 1.61; prediction interval 0.75, 2.33). |
|  |  |  |  |  |  |  |  |  |  |  |
| Clayborne et al 2019 | Depression | Psychosocial outcomes (educational attainment--whether completed secondary education, income, employment, pregnancy/ parenthood, marital and relationship status, social support and loneliness) | English | 1980 (when the DSM-III definition of major depressive disorder was introduced) to 2017 | Prospective cohorts and case-control studies of depression in adolescence (10-19 yrs) and outcomes measured in adulthood with a minimum of 12 months between exposure measurement and outcome. | 14/31 | USA (9), Canada (3), New Zealand (2) | 63 to 14,232 (531) | Individuals with depression aged 10-19 (diagnosed as major depressive disorder according to DSM or ICD or the following via self-report or diagnostic interviews: depressive disorder, major depressive disorder, major depression or clinically significant depressive symptoms) *vs* individuals without | Odds ratio (95% CI) for not completing secondary school in those with *vs* those without depression: 1.97 (1.73, 2.24). When excluding studies that did not adjust for covariates: 1.42 (0.91, 2.20). When excluding lower quality studies: 1.68 (1.12, 2.52). |
|  |  |  |  |  |  |  |  |  |  |  |
| Wickersham et al 2021 | Depression | Educational attainment | English | Database inception to 2018 | Studies of a diagnosis of depression or depression symptoms measured using a standardised diagnostic measure or named instrument and educational attainment measured using academic or administrative records. Studies had to be longitudinal in design, of 4-18 year-olds in counties with compulsory education. | 31/31 (of which, 22 were included in meta-analysis) | USA (16), China (6), UK (5), Sweden (1), Canada (1), Australia (1), France (1) | Of all 31 studies: 129 to 7,276 (498). Of 22 in meta-analysis: 129 to 7,276 (372) | Healthy controls | In meta-analysis, depression was slightly negatively associated with poorer attainment with a pooled Fisher z of -0.19 (95% CI: -0.22, -0.16), equivalent to a pooled correlation coefficient of -0.19, indicating that depression symptoms accounted for 3.6% of the variance in attainment. In 15 of these studies, results remained statistically significant after adjusting for confounders. Eight of 12 studies that adjusted for attainment at an earlier time point also remained significant, indicating lower likelihood of reverse causation. Analyses of effect modification by gender and ethnicity were inconclusive. |
|  |  |  |  |  |  |  |  |  |  |  |
| Puka et al 2019 | Epilepsy | Educational attainment, defined by completion of secondary, post-secondary or higher education (other adult outcomes were also studied) | English | 1987 to 2018 | Childhood-onset epilepsy and a later outcome. | 18/45 on completion of secondary education | Canada (4), Finland (3), USA (2), Germany (2), Norway (1), UK (1), Sweden (1), Australia (1), Denmark (1), Netherlands (1), Japan (1) | 16 to 11,400 (77) | Studies either had healthy controls, comparisons with population norms or no control/ comparisons | Overall, 73% (95% CI 64%, 82%) of children with epilepsy completed secondary school. Win 10 studies with comparison groups, children with epilepsy did worse in 7 studies and the same in 3. When examining by prognosis, 39% (8%, 76%) of children with epilepsy completed secondary schooling (4 studies; worse than controls in all 3 studies with control groups). Among those with good prognosis, 77% (63%, 88%) completed secondary schooling (9 studies; worse in 2 studies with control groups but the same in 3 studies). 98% (86%, 100%) of children who had resective surgery in childhood completed secondary schooling (3 studies, no comparison groups). |
|  |  |  |  |  |  |  |  |  |  |  |
| Wo et al 2017 | Epilepsy | Academic achievement measured by standardised, objective measures. | English | Database inception to 2015 | Cross-sectional and longitudinal studies with children aged 5-18 years of IQ >= 70, attending regular school with or without a control group. Studies were excluded if they were of children with comorbidities such as autism, intellectual disability or learning difficulties. | 20/20 | USA (13), Netherlands (3), Brazil (2), Jamaica (1), Turkey (1) | 42 to 225 (83) | Control groups or comparisons with population norms | Fourteen of 20 studies reported that children with epilepsy had lower achievement than controls/population norms whereas the remaining studies did not. There was heterogeneity in study design (e.g., instruments used, timing of measurements) which make comparisons difficult. |
|  |  |  |  |  |  |  |  |  |  |  |
| Lah et al 2017 | Epilepsy (temporal lobe) | Reading ability | English | Database inception to 2016 | Empirical research examining reading ability in children with temporal lobe epilepsy either on a standardised test or by comparing children with TLE vs a control group. | 6/6 | Canada (5), France (1) | 19 to 60 (37) | Either normative data (5 studies) or control group (1 study) | There was evidence from two studies that children with temporal lobe epilepsy have lower reading accuracy and comprehension, though in one study this was restricted to left TLE post-surgery. Reading accuracy was not lower than controls in the one study with a control group. |
|  |  |  |  |  |  |  |  |  |  |  |
| Caird et al 2013 | Obesity | Attainment (e.g., grade point average) | English | 1997 to 2008 | Children aged 6-16 in high income countries. Quantitative only. | 29/29 | USA (23), UK (2), South Korea (1), Finland (1), Canada (1), Iceland (1) | 259 to 60,252 (3,500) | Comparisons were between children with higher/lower BMI or between those with and without obesity or overweight. | Most studies reported a very small correlation between higher BMI/overweight/obesity and lower attainment. In many instances, adjusting for factors such as socioeconomic position accounted for the association. The authors conclude that, if there is a causal link between obesity and attainment, it is not likely of clinical significance. Some studies reported higher attainment in those with overweight/obesity. |
|  |  |  |  |  |  |  |  |  |  |  |
| Martin et al 2017 | Obesity | Achievement (standardised test scores, teacher- or self-reported grades or average attainment during compulsory education) | English | Database inception to 2017 | Longitudinal studies of children aged 3-18 years with measured or self-reported weight, body fat or waist circumference (or BMI-derived status such as obese). | 31/31 (reported in 30 articles) | USA (17), Taiwan (2), Australia (2), Canada (2), UK (2), Netherlands (2), Germany (1), Thailand (1), Peru (1) | 405 to 21,260 (3,362) | Comparisons were with children not overweight/obese or by continuous measures of percentage body fat, BMI z scores or BMI | Obesity was negatively associated with maths achievement among adolescent girls, mediated partly by psychosocial experiences such as bullying and cognitive abilities involving executive functions. There was no clear evidence for other subjects. There was generally no association between obesity and achievement in younger children. |
|  |  |  |  |  |  |  |  |  |  |  |
| Santana et al 2017 | Obesity | Academic performance | English | Database inception to 2016 | Cross-sectional and longitudinal studies in students <20 years of age examining obesity and an objective measure of academic performance | 34/34 | USA (16), Netherlands (2), Colombia (2), Canada (2), Portugal (1), Finland (1), Taiwan (1), Iran (1), Denmark (2), Italy (1), Spain (1), Chile (1), UK (1), Korea (1), Kuwait (1), Japan (1) | 37 to 18,746 (1808) | Comparisons with healthy weight peers or gradations in BMI | 11/23 cross-sectional and 4/11 longitudinal studies reported an association between obesity and academic performance. However, none of the four cross-sectional studies of low risk of bias reported an association after adjusting for confounders and only 8/16 medium risk cross-sectional studies did so. Of the four longitudinal studies with low risk of bias, two reported an association such that those who were or became obese had lower performance than healthy-weight peers. In one of these studies, the association was only observed in girls. Only one medium-risk longitudinal study reported an association. The level of evidence is therefore uncertain. |
|  |  |  |  |  |  |  |  |  |  |  |
| Segal et al 2021 | Obesity | Human capital (including educational attainment measured either using standardised tests, grade point average or educational attainment) | English | 1980 to 2019 | Studies had to examine childhood obesity or overweight (without comorbid conditions) in high income countries, be longitudinal and use methods that account for reverse causation and unobserved confounding (i.e., quasi-experimental designs). | 18/19 | USA (11), UK (2), Australia (2), Taiwan (2), Canada (1) | 409 to 21,260 (4,914) | Continuous BMI or categories of weight | Obesity/overweight, especially at older ages (12+ years) was associated with poorer achievement/attainment. Girls experienced larger negative effects than boys. |
| Milton et al 2006 | Type 1 diabetes | Academic achievement (tests or grades or completion of education) and school absence | English | 1975 to 2002 | Industrialised countries with participants aged less than 18 at time of diabetes type 1 onset. | 11/20 | USA (5), Canada (3), UK (3) | 54 to 563 (116) | Siblings or non-related controls | Although four studies found that children with diabetes were more likely to be absent from school, almost all studies examining attainment found either no difference in attainment between children with and without diabetes or better attainment in those with. However, studies did not generally consider aspects of disease. One study found worse attainment in children with diabetes onset before age 7 and a number of studies found that better/worse metabolic control was associated with better/worse attainment. Most studies were case-control. |
|  |  |  |  |  |  |  |  |  |  |  |
| Oakley et al 2020 | Type 1 diabetes | High-stakes testing at the end of compulsory education | English | 2004 to 2019 | Observational study or RCT with individuals undertaking high-stakes testing at the end of compulsory education. Comparisons with a representative population control group. | 2/2 | Sweden (2) | 11,995 and 1,336,126 | Population-representative control group | Both studies were of Swedish population registries of individuals born between 1972 and 1985, both of which adjusted for year of birth, gender, school year and level of parental education. One study also adjusted for parental income and country of origin and the other adjusted for parity. Both studies found a weak negative association between diabetes and overall attainment at age 16. The association was stronger for sports/athletics. |
|  |  |  |  |  |  |  |  |  |  |  |
