# Supplementary material for: Umbrella systematic review finds limited evidence that school absence explains the association between chronic health conditions and lower academic attainment
Source: Front Public Health. 2023 Jun 9;11:1122769. doi: 10.3389/fpubh.2023.1122769 (PMC10288991; doi:10.3389/fpubh.2023.1122769)
Supplement: Supplementary file 4 [file Table_4.DOCX]

**Supplementary File 4 – risk of bias assessment**

Supplementary Table 4.1. Assessment of risk of bias of the 27 reviews using the Risk of Bias in Systematic Reviews (ROBIS) tool

| Review |  | Condition | 1. Study eligibility | 2. Identification & selection | 3. Data collection & appraisal | 4. Synthesis | Overall risk of bias |
| --- | --- | --- | --- | --- | --- | --- | --- |
| Generic or multiple | McKinley Yoder & Cantrell 2019 | Generic / multiple | ☺ | ☹ | ☹ | ☺ | ☹ |
|  | Hale et al 2015 | Generic / multiple | ☹ | ☹ | ☹ | ☺ | ☹ |
|  | Moser et al 2013 | Generic / multiple | ☹ | ☹ | ☹ | ☺ | ☹ |
|  | Glinianaia et al 2021 | Major structural congenital anomalies | ☹ | ☺ | ☺ | ☺ | ☹ |
|  | Esch et al 2014 | Mental disorders | ☺ | ☹ | ☹ | ☺ | ☹ |
| Condition-specific | Milton et al 2004 | Asthma | ☹ | ☹ | ☺ | ☺ | ☹ |
|  | Schneider 2020 | Asthma | ☹ | ☹ | ☹ | ☺ | ☹ |
|  | Polderman et al 2010 | Attention problems including ADHD | ☺ | ☹ | ☺ | ☺ | ☹ |
|  | Schulte et al 2019 | Cancer: CNS tumour survivors | ☹ | ☹ | ☺ | ☺ | ☹ |
|  | Langevald et al 2002 | Cancer: Survivors of childhood cancer | ☹ | ☹ | ☹ | ☺ | ☹ |
|  | Molcho et al 2019 | Cancer: Survivors of childhood cancer | ☹ | ☹ | ☺ | ☺ | ☹ |
|  | Saatci et al 2020 | Cancer: Survivors of childhood cancer | ☺ | ☺ | ☺ | ☺ | ☺ |
|  | Chen et al 2018 | Chronic kidney disease | ☺ | ☺ | ☺ | ☺ | ☺ |
|  | Alsaggaf & Coyne 2020 | Chronic pain | ☹ | ☹ | ☹ | ☺ | ☹ |
|  | Ragnarsson et al 2020 | Chronic pain | ☹ | ☺ | ☺ | ☺ | ☹ |
|  | Cocomello et al 2021 | Congenital heart disease | ☺ | ☹ | ☺ | ☺ | ☹ |
|  | Clayborne et al 2019 | Depression | ☹ | ☺ | ☺ | ☺ | ☹ |
|  | Wickersham et al 2021 | Depression | ☹ | ☺ | ☺ | ☺ | ☹ |
|  | Puka et al 2019 | Epilepsy | ☹ | ☹ | ☺ | ☺ | ☹ |
|  | Wo et al 2017 | Epilepsy | ☹ | ☹ | ☺ | ☺ | ☹ |
|  | Lah et al 2017 | Epilepsy (temporal lobe) | ☹ | ☹ | ☺ | ☺ | ☹ |
|  | Caird et al 2013 | Obesity | ☹ | ☹ | ☺ | ☺ | ☹ |
|  | Martin et al 2017 | Obesity | ☹ | ☺ | ☺ | ☺ | ☹ |
|  | Santana et al 2017 | Obesity | ☹ | ☺ | ☺ | ☺ | ☹ |
|  | Segal et al 2021 | Obesity | ☹ | ☹ | ☺ | ☺ | ☹ |
|  | Milton et al 2006 | Type 1 diabetes | ☹ | ☹ | ☹ | ☺ | ☹ |
|  | Oakley et al 2020 | Type 1 diabetes | ☹ | ☺ | ☺ | ☺ | ☹ |

☺ = low risk; ☹ = high risk
